# Association of hemodialysis vascular access type with dialysis adequacy and access-related outcomes in patients receiving maintenance hemodialysis

**DOI:** 10.3389/fmed.2026.1890240

**Published:** 2026-10-07

**Authors:** Ying Zhu, Jie Zhao, Bing Liu

**Affiliations:** 1 Department of Nephrology Blood Purification Center, The Affiliated Wuxi People's Hospital of Nanjing Medical University (Wuxi People's Hospital, Wuxi Medical Center, Nanjing Medical University), Wuxi, Jiangsu, China; 2 Hemodialysis Unit, Huai'an Hospital of Huai’an City (Huai'an Clinical Medical College of Jiangsu University), Huai'an, Jiangsu, China; 3 Department of Nephrology, Yangxin County People’s Hospital, Huangshi, Hubei, China

**Keywords:** access-related complications, hemodialysis, residual kidney function, single-pool Kt/V, vascular access

## Abstract

**Background:**

Vascular access type may be associated with the delivered hemodialysis dose and access-related morbidity; however, nonrandom access selection introduces confounding. We evaluated the associations of arteriovenous fistula (AVF), arteriovenous graft (AVG), and tunneled central venous catheter (CVC) use with dialysis adequacy and access-related outcomes.

**Methods:**

This retrospective study included 285 adults whose index access was first successfully used between 2020 and 2023, with follow-up through 2024. Monthly single-pool Kt/V (spKt/V) measurements during the first 6 months were analyzed using mixed-effects models. Recurrent complications were reported as rates per 100 access-years. Cox regression and Kaplan–Meier methods were used to evaluate the first composite complication and permanent access failure, respectively. Models were adjusted for prespecified baseline factors, including a history of urgent-start dialysis, previous access attempts, vascular mapping, and residual kidney urea clearance; the interval from access creation or placement to first successful use was examined in sensitivity analyses.

**Results:**

The AVF, AVG, and CVC groups included 156, 67, and 62 patients, respectively, and contributed 1,632 adequacy measurements. Compared with CVC use, adjusted monthly spKt/V was higher with AVF use (*β* = 0.23, 95% confidence interval [CI] 0.17–0.29; *P* < 0.001) and AVG use (*β* = 0.11, 95% CI: 0.04–0.18; *P* = 0.002). Recurrent composite-complication rates were 8.84, 26.60, and 54.69 per 100 access-years, respectively (*P* < 0.001). AVF use was associated with a lower hazard of the first composite complication than CVC use (hazard ratio [HR] = 0.39, 95% CI: 0.23–0.66; *P* < 0.001), whereas the estimate for AVG was not statistically significant. Among accesses that had reached successful clinical use, 12-month observed access survival from first successful use was 94.0%, 84.7%, and 57.4% in the AVF, AVG, and CVC groups, respectively.

**Conclusions:**

AVF use was associated with higher dialysis adequacy and a lower burden of access-related complications than CVC use. Among accesses that had reached successful clinical use, AVFs showed longer observed access survival than AVGs and CVCs. Access selection should integrate clinical urgency, vascular anatomy, anticipated access use, comorbidity, residual kidney function, and patient priorities.

## 1 Introduction

Chronic kidney disease is a major contributor to the global burden of noncommunicable disease; progression to kidney failure often necessitates kidney replacement therapy and is accompanied by substantial mortality, cardiovascular events, and healthcare resource use (1). The burden of chronic kidney disease continues to increase in low- and middle-income countries, and differences in dialysis availability, continuity, and quality further widen disparities in patient outcomes (2). Cardiovascular disease is a major driver of death and hospitalization among patients with kidney failure, with risks compounded by diabetes, vascular disease, inflammation, and dialysis-related factors (3, 4). Reliable vascular access is essential for the stable delivery of maintenance hemodialysis. A native arteriovenous fistula (AVF), an arteriovenous graft (AVG), and a tunneled central venous catheter (CVC) differ in their creation, immediate usability, and complication profiles; access function also affects achievable blood flow, recirculation, and treatment interruption (5). The use of AVFs, AVGs, and tunneled CVCs, as well as their funding arrangements, varies markedly across regions, indicating that access selection is influenced by both resource availability and health-system factors (6). Real-world data show associations of access type with mortality and access-related outcomes, but age, diabetes, cardiovascular disease, urgent-start dialysis, and previous access failure also influence both access selection and prognosis (7). In older patients or those with multimorbidity, simply ranking access types may overlook life expectancy, vascular anatomy, residual kidney function, and the patient's treatment goals (8). Contemporary vascular access management emphasizes a kidney failure life-plan framework that balances durability, timing of creation, complications, future treatment pathways, and patient preferences (9). The timing of access creation is closely linked to kidney failure risk assessment: late creation increases catheter exposure, whereas premature creation may result in unnecessary surgery and an unused access (10). This study compared the associations of three index access types with repeated dialysis adequacy measurements during the first 6 months, complication rates according to access-exposure time, and access survival from first successful use, and assessed the robustness of the findings using multivariable, propensity-score, and competing-risk analyses (11).

## 2 Materials and methods

### 2.1 Study design, participants, and time zero

This retrospective study used electronic medical records and a dialysis information system. Records from January 1, 2019, to December 31, 2024, were reviewed. Adults whose time zero occurred between January 1, 2020, and December 31, 2023, were included, ensuring that every participant had an administrative follow-up window of at least 12 months by the study end date; actual observation could end earlier because of permanent access failure, death, kidney transplantation, transfer or loss to follow-up, or planned conversion without access failure. Time zero was defined as the date on which the index access first successfully supported the prescribed dialysis treatment. For an AVF or AVG, the entire dialysis session had to be completed with two-needle cannulation during the same treatment; for a tunneled CVC, the first complete dialysis session had to be delivered through that catheter. The access used at time zero was fixed as the index access. The inclusion criteria were age ≥18 years, receipt of thrice-weekly maintenance hemodialysis, and an index access that could be clearly classified as an AVF, AVG, or tunneled CVC. The exclusion criteria were acute kidney injury, peritoneal dialysis or home hemodialysis, alternating use of two access types at time zero without an identifiable primary access, missing key access dates or outcome data, and an expected survival of <6 months. All 285 patients were included in the access-outcome analysis. At least one valid measurement was required for the monthly adequacy analysis, and all 285 patients met this requirement. Patients who underwent access conversion after index access failure remained in the study; the failure date was recorded as the event date for the original index access, and data after conversion were no longer attributed to the original index access.

### 2.2 Index access, previous access history, vascular mapping, and follow-up

Follow-up began at time zero and continued until the earliest of permanent index access failure, death, kidney transplantation, transfer or loss to follow-up, planned conversion of a CVC without failure to a mature AVF or AVG, or the study end date. The primary Kaplan–Meier analysis treated death, kidney transplantation, transfer or loss to follow-up, planned conversion without failure, and the study end date as censoring events; the competing-risk analysis treated death, kidney transplantation, and planned conversion as competing events. Because time zero was defined as the first successful use of the index access, the age of the index access was 0 at time zero. The interval from access creation or placement to first successful use and any previous AVF or AVG creation attempts were also recorded. Preoperative vascular mapping documented the patency, continuity, compressibility, outflow tract, and diameters of the target artery and vein. Suitability for a native AVF was defined as the absence of significant stenosis or thrombosis, a target arterial diameter ≥2.0 mm, a venous diameter ≥2.5 mm, and overall confirmation of suitability by the vascular access team (12). Urgent-start dialysis was defined as initiation of the first maintenance hemodialysis session within 14 days after the decision for long-term dialysis when no AVF or AVG was immediately usable; this variable represented the historical status at dialysis initiation.

### 2.3 Dialysis prescription and achieved treatment parameters

All patients underwent thrice-weekly high-flux hemodialysis using bicarbonate dialysate. The routine prescribed treatment duration was 240 min, and the dialysate flow rate was 500 mL/min. The routinely achievable blood pump flow ranges at our center were 300–400 mL/min for AVFs or AVGs and 250–350 mL/min for tunneled CVCs; these ranges reflected the flow rates achievable under constraints related to access function, extracorporeal circuit pressures, and equipment safety. The actual blood pump flow for each treatment was titrated according to access function, arterial and venous pressures, recirculation, hemodynamic tolerance, and equipment alarms. Prescribed duration, actual treatment duration, median intradialytic blood pump flow, dialyzer membrane area, access recirculation, missed dialysis, interdialytic weight gain, and ultrafiltration rate were recorded. A missed dialysis treatment was defined as a scheduled treatment that was not completed and was not made up within 48 h. Placement, maintenance, and functional assessment of tunneled catheters followed current catheter-management principles (13).

### 2.4 Measurement of dialysis adequacy

Dialysis adequacy was measured during a prespecified midweek treatment each month for 6 consecutive months beginning with the first successful use of the index access. Predialysis blood urea nitrogen was sampled from the arterial needle or the arterial lumen of the catheter. For catheter sampling, the lock solution was completely aspirated and discarded before blood collection; all predialysis samples were obtained before saline infusion, heparin administration, and initiation of the blood pump. Postdialysis blood sampling used the slow-flow method: the blood pump flow was reduced to 100 mL/min and maintained for 15 s before sampling from the arterial bloodline to minimize bias from access and cardiopulmonary recirculation (14). Single-pool Kt/V (spKt/V) was calculated using the second-generation Daugirdas equation: spKt/V = −ln(R − 0.008t) + (4 − 3.5R) × UF/W, where R is the ratio of postdialysis to predialysis blood urea nitrogen, t is the actual treatment duration (h), UF is the ultrafiltration volume (L), and W is the postdialysis body weight (kg). The urea reduction ratio (URR) was calculated as [(predialysis blood urea nitrogen − postdialysis blood urea nitrogen)/predialysis blood urea nitrogen] × 100%.

The primary adequacy outcome was the repeated monthly spKt/V measurements during the first 6 months. Patient-specific 6-month mean spKt/V and mean URR were used only for descriptive purposes. Monthly spKt/V ≥ 1.2 represented the minimum delivered dialysis dose, spKt/V ≥1.4 represented the target dose, and URR ≥65% was used as a supportive threshold; the three measures were analyzed as separate outcomes (15).

### 2.5 Residual kidney function and fluid management

Baseline residual kidney function was measured within 30 days before or after the index date using a standardized 24 h interdialytic urine collection. Collection began after completion of a dialysis session and continued for 24 h until the same time on the following day. The actual collection duration and total urine volume were recorded, and plasma urea nitrogen was measured at the beginning and end of the collection period. Residual kidney urea clearance (Kru) was calculated as (urine urea nitrogen concentration × urine volume)/(mean plasma urea nitrogen concentration during the collection period × actual collection duration) and standardized to a body surface area of 1.73 m^2^. spKt/V and URR characterized urea clearance delivered by dialysis, whereas Kru separately characterized residual native-kidney clearance. The 24 h urine volume, urine volume ≥200 mL/d, patient-specific mean interdialytic weight gain as a percentage of postdialysis target weight during the first 6 months, and mean ultrafiltration rate were recorded to assess residual clearance and volume management (16).

### 2.6 Definitions of access-related outcomes

Access-related infection was defined as a local or systemic infection requiring systemic antimicrobial therapy and clinically judged to originate from the index access (17). CVC-related bloodstream infection was defined by compatible systemic manifestations, no other definite source of infection, and either of the following microbiological criteria: isolation of the same pathogen from blood cultures drawn from the catheter lumen and a peripheral vein, with a differential time to positivity of ≥2 h in favor of the catheter specimen; or isolation of the same pathogen from the catheter-tip culture and a blood culture after catheter removal (18). All CVC-related bloodstream infection events reported in Table 3 met these criteria. Thrombosis was defined as an intraluminal access thrombus confirmed by clinical, imaging, or interventional records that caused reduced flow, dialysis failure, or the need for treatment. Intervention-requiring stenosis was defined as hemodynamically significant stenosis confirmed by imaging or functional assessment and treated using an endovascular or surgical procedure (19). The first occurrence of infection, thrombosis, or intervention-requiring stenosis constituted the first composite complication. Permanent access failure was not included in this composite outcome and was analyzed separately as the access-survival outcome. Permanent access failure was defined as inability of the index access to continue supporting the prescribed dialysis treatment, necessitating permanent abandonment or replacement.

### 2.7 Statistical analysis

Continuous variables were presented as mean ± standard deviation (SD) or median [interquartile range (IQR)], as appropriate for their distributions, and categorical variables as *n* (%). Three-group comparisons used one-way analysis of variance, the Kruskal–Wallis test, the *χ*^2^ test, or Fisher's exact test. Monthly spKt/V was analyzed using a linear mixed-effects model with a patient-specific random intercept. Month was centered at 0 at the first measurement, and the residual correlation structure was selected as first-order autoregressive [AR(1)] according to the Akaike information criterion. Fixed effects included access type, month, access type × month, and prespecified pretreatment confounders. The interval from access creation or placement to first successful use was jointly determined by access type and the maturation process and was therefore not included as a primary pretreatment confounder in the main model; an extended model assessed robustness by including log₂(interval + 1 d). Actual treatment duration, blood pump flow, recirculation, dialyzer membrane area, and missed dialysis in the current month were treated as treatment-delivery factors and included only in a supplementary explanatory model. Monthly spKt/V ≥1.2, spKt/V ≥1.4, and URR ≥65% were each analyzed using a generalized linear mixed model with a patient-specific random intercept. Recurrent complications were analyzed using patient-level negative binomial regression, with each patient's cumulative event count during follow-up as the outcome, log(individual access-exposure years) as the offset, and robust (sandwich) standard errors. Incidence rates were reported as events per 100 access-years with exact Poisson 95% confidence intervals (CIs). The first composite complication was analyzed using a Cox proportional hazards model, and permanent access failure was analyzed using Kaplan–Meier methods and the log-rank test. Of 1,710 theoretically expected monthly adequacy measurements, 78 were unavailable; the primary mixed model used restricted maximum likelihood to incorporate all available outcomes. Stabilized inverse-probability-of-observation weights were also constructed. The observation model included baseline covariates, adequacy in the preceding month, previous hospitalization, and previous access events, and weights were truncated at the 1st and 99th percentiles. Body mass index (BMI) was missing for 2 patients, albumin for 3, vascular mapping for 16, Kru for 11, actual treatment duration for 4, blood pump flow for 3, and recirculation for 5; access type, clinical outcomes, and event or censoring times had no missing data. Covariates were imputed using multiple imputation by chained equations with 20 imputations. The imputation model included all baseline variables, access type, outcome events, follow-up time, and the Nelson-Aalen cumulative hazard. Predictive mean matching was used for continuous variables and the corresponding regression models for categorical variables; estimates were pooled across imputed datasets according to Rubin's rules, whereas outcome and follow-up data retained their original recorded values. Three-category propensity scores were estimated using multinomial logistic regression and applied for overlap weighting; an absolute standardized difference <0.10 after weighting indicated balance. Model diagnostics included residual distribution, homoscedasticity, Schoenfeld residuals, the variance inflation factor (VIF), the Harrell C statistic, and weighted absolute standardized differences. All tests were two-sided; *P* < 0.05 was considered statistically significant.

## 3 Results

### 3.1 Patient selection and baseline characteristics

Records for 367 patients were screened, 82 were excluded, and 285 were included: 156 in the AVF group, 67 in the AVG group, and 62 in the CVC group. Age, sex, BMI, cause of end-stage kidney disease, hemoglobin, and albumin did not differ significantly among the three groups (all *P* > 0.05). The CVC group had higher proportions of diabetes and cardiovascular disease, shorter dialysis vintage, more frequent histories of urgent-start dialysis and previous permanent-access creation attempts, a greater proportion of patients with preserved urine output ≥200 mL/d, and a lower proportion with vascular mapping suitable for a native AVF. The interval from access creation or placement to first successful use was longest in the AVF group, intermediate in the AVG group, and shortest in the CVC group. The CVC group had greater 24 h urine volume and Kru than the other groups (both *P* < 0.05) (Table 1; Figure 1).

**Table 1:** Baseline clinical characteristics of the three groups.

Variable	AVF group (*n* = 156)	AVG group (*n* = 67)	CVC group (*n* = 62)	*P* value
Age, years	58.3 ± 14.5	59.2 ± 13.8	59.1 ± 14.6	0.883
Male sex, *n* (%)	94 (60.3)	39 (58.2)	37 (59.7)	0.960
Body mass index, kg/m^2^	24.6 ± 4.2	24.8 ± 4.5	25.1 ± 4.3	0.737
Cause of end-stage kidney disease (overall)				0.163
Diabetic kidney disease, *n* (%)	43 (27.6)	20 (29.9)	30 (48.4)	
Chronic glomerulonephritis, *n* (%)	48 (30.8)	19 (28.4)	14 (22.6)	
Hypertensive nephropathy, *n* (%)	38 (24.4)	16 (23.9)	10 (16.1)	
Other, *n* (%)	27 (17.3)	12 (17.9)	8 (12.9)	
Diabetes, *n* (%)	51 (32.7)	24 (35.8)	36 (58.1)	0.002
Cardiovascular disease, *n* (%)	40 (25.6)	19 (28.4)	28 (45.2)	0.017
Dialysis vintage, months	36 [18–52]	32 [16–46]	18 [10–34]	<0.001
History of urgent-start dialysis, *n* (%)	12 (7.7)	8 (11.9)	24 (38.7)	<0.001
≥1 previous AVF or AVG creation attempt, *n* (%)	24 (15.4)	29 (43.3)	27 (43.5)	<0.001
Vascular mapping suitable for native AVF, *n*/*N* (%)	139/150 (92.7)	28/62 (45.2)	17/57 (29.8)	<0.001
Interval from access creation or placement to first successful use, d	62 [49–82]	25 [18–34]	1 [0–2]	<0.001
24 h urine volume, mL	80 [0–260]	120 [0–300]	260 [60–650]	<0.001
Kru, mL/min/1.73 m^2^	0.3 [0–1.1]	0.4 [0–1.3]	0.9 [0.2–2.0]	<0.001
24 h urine volume ≥200 mL, *n* (%)	52 (33.3)	25 (37.3)	34 (54.8)	0.013
Hemoglobin, g/dL	10.8 ± 1.4	10.6 ± 1.5	10.4 ± 1.6	0.179
Albumin, g/dL	3.8 ± 0.4	3.7 ± 0.5	3.7 ± 0.5	0.174

Continuous variables are presented as mean ± standard deviation (SD) or median [first quartile, third quartile], as appropriate, and categorical variables as number (percentage). Continuous variables were compared among the three groups using one-way analysis of variance or the Kruskal–Wallis test, and categorical variables using the Pearson *χ*^2^ test or Fisher's exact test. The *P* value for cause of end-stage kidney disease represents the overall comparison. Vascular mapping, residual kidney urea clearance, and albumin were analyzed using the number of available observations; missing covariates were handled using multiple imputation by chained equations. All *P* values are two-sided.

n, number of patients; N, total number of patients with available data; AVF, arteriovenous fistula; AVG, arteriovenous graft; CVC, tunneled central venous catheter; BMI, body mass index; Kru, residual kidney urea clearance standardized to a body surface area of 1.73 m^2^; IQR, interquartile range; SD, standard deviation.

**Figure 1:**
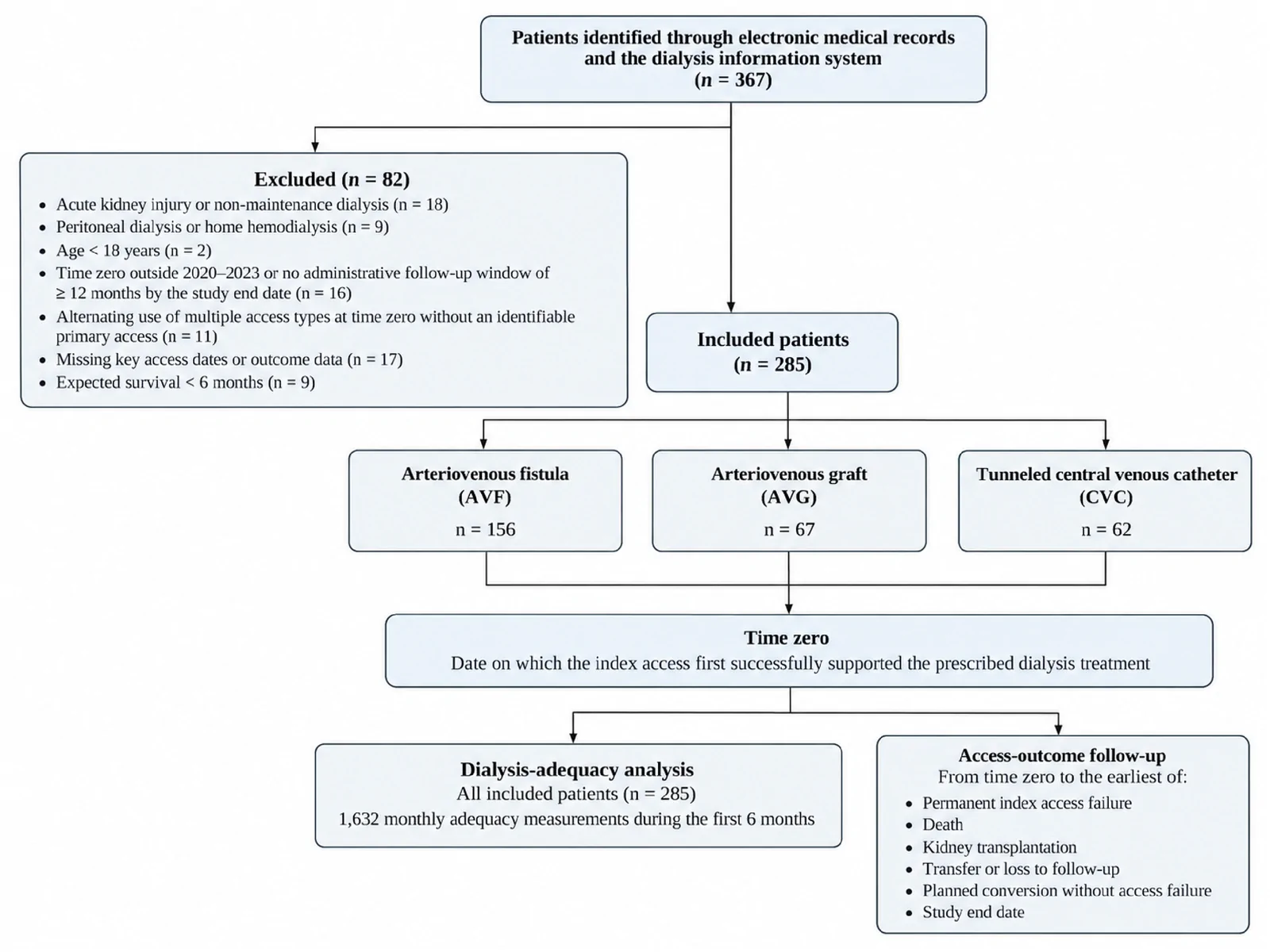
Patient screening, index vascular access assignment, and follow-up flow.

A total of 367 patients were identified through electronic medical records and the dialysis information system. According to prespecified mutually exclusive exclusion criteria, 82 patients were excluded: acute kidney injury or non-maintenance dialysis, *n* = 18; peritoneal dialysis or home hemodialysis, *n* = 9; age <18 years, *n* = 2; time zero outside 2020–2023 or no administrative follow-up window of ≥12 months by the study end date, *n* = 16; alternating use of multiple access types at time zero without an identifiable primary access, *n* = 11; missing key access dates or outcome data, *n* = 17; and expected survival <6 months, *n* = 9. The remaining 285 patients were included in the access-outcome analysis and all were also included in the dialysis-adequacy analysis, contributing 1,632 monthly adequacy measurements. Patients were assigned according to the index access used at time zero into the arteriovenous fistula group (AVF, *n* = 156), arteriovenous graft group (AVG, *n* = 67), or tunneled central venous catheter group (CVC, *n* = 62). Time zero was defined as the date on which the index access first successfully supported the prescribed dialysis treatment. Follow-up began at time zero and continued until the earliest occurrence of permanent index access failure, death, kidney transplantation, transfer or loss to follow-up, planned conversion without access failure, or the study end date. AVF, arteriovenous fistula; AVG, arteriovenous graft; CVC, central venous catheter.

### 3.2 Primary indications for CVC use

The primary indications for CVC use, in descending frequency, were urgent-start dialysis; vascular anatomy or systemic conditions unsuitable for timely AVF or AVG creation or use; repeated failure of previous permanent access or exhaustion of usable sites; waiting for a previously created AVF or AVG to mature; and informed patient choice or other reasons (Supplementary Table S1).

### 3.3 Dialysis adequacy, treatment parameters, and fluid management

The number of monthly adequacy measurements obtained during the first 6 months was lower than the theoretical maximum. The proportion of patients who completed all 6 monthly measurements was highest in the AVF group, intermediate in the AVG group, and lowest in the CVC group (*P* < 0.05). Unavailable measurements were mainly attributable to index access failure or planned conversion, hospitalization or failure to complete the prespecified midweek treatment, and sampling or laboratory procedures that did not meet prespecified requirements. Patient-specific mean spKt/V, mean URR, and the proportions of monthly measurements meeting spKt/V ≥1.2, spKt/V ≥1.4, and URR ≥65% were all highest in the AVF group, intermediate in the AVG group, and lowest in the CVC group (all *P* < 0.001). Actual blood pump flow and actual treatment duration followed the same pattern. Access recirculation was greater and missed dialysis was more frequent in the CVC group, whereas dialyzer membrane area was similar among the three groups (*P* > 0.05). The CVC group had a lower interdialytic weight-gain percentage and a lower ultrafiltration rate (both *P* < 0.05), consistent with its greater residual urine volume (Table 2; Figure 2).

**Table 2:** Dialysis adequacy, achieved treatment parameters, and fluid management during the first 6 months.

Variable	AVF group	AVG group	CVC group	*P* value
Valid monthly measurements, no.	918	384	330	—
Patient-specific 6-month mean spKt/V	1.52 ± 0.21	1.38 ± 0.19	1.25 ± 0.23	<0.001
Patient-specific 6-month mean URR, %	72.5 ± 6.2	68.3 ± 7.1	63.8 ± 8.5	<0.001
Monthly spKt/V ≥1.2, no./total no. (%)	862/918 (93.9)	316/384 (82.3)	198/330 (60.0)	<0.001
Monthly spKt/V ≥1.4, no./total no. (%)	653/918 (71.1)	178/384 (46.4)	80/330 (24.2)	<0.001
Monthly URR ≥65%, no./total no. (%)	818/918 (89.1)	264/384 (68.8)	149/330 (45.2)	<0.001
Actual blood pump flow, mL/min	352 ± 45	328 ± 52	285 ± 38	<0.001
Actual treatment duration, min	238 ± 6	236 ± 8	232 ± 12	<0.001
Dialyzer membrane area, m^2^	1.82 ± 0.16	1.80 ± 0.17	1.79 ± 0.18	0.434
Access recirculation, %	1.2 ± 1.8	3.1 ± 2.4	8.3 ± 3.2	<0.001
At least 1 missed dialysis treatment during the first 6 months, *n* (%)	12 (7.7)	8 (11.9)	13 (21.0)	0.022
Mean interdialytic weight gain/postdialysis target weight, %	3.1 ± 0.9	3.0 ± 0.9	2.6 ± 0.8	<0.001
Mean ultrafiltration rate, mL/kg/h	8.2 ± 2.4	8.0 ± 2.5	7.1 ± 2.3	0.010
Completed all 6 monthly measurements, *n* (%)	145 (92.9)	60 (89.6)	47 (75.8)	0.002

Continuous measures are patient-specific means during the first 6 months; monthly threshold-attainment rates use the number of valid monthly measurements as the denominator. Of 1,710 theoretically expected measurements, 1,632 were obtained, and 252 patients completed all 6 monthly measurements. The remaining 78 unavailable measurements comprised 38 due to access failure or conversion, 24 due to hospitalization or noncompletion of the prespecified treatment, and 16 due to sampling or laboratory procedures that did not meet prespecified requirements. Continuous variables were compared among the three groups using one-way analysis of variance or the Kruskal–Wallis test, and categorical variables using the Pearson *χ*^2^ test. Monthly threshold-attainment rates were compared using generalized linear mixed-effects models with a patient-specific random intercept and logit link. All *P* values are two-sided.

AVF, arteriovenous fistula; AVG, arteriovenous graft; CVC, tunneled central venous catheter; spKt/V, single-pool Kt/V; URR, urea reduction ratio; IQR, interquartile range; SD, standard deviation.

**Figure 2:**
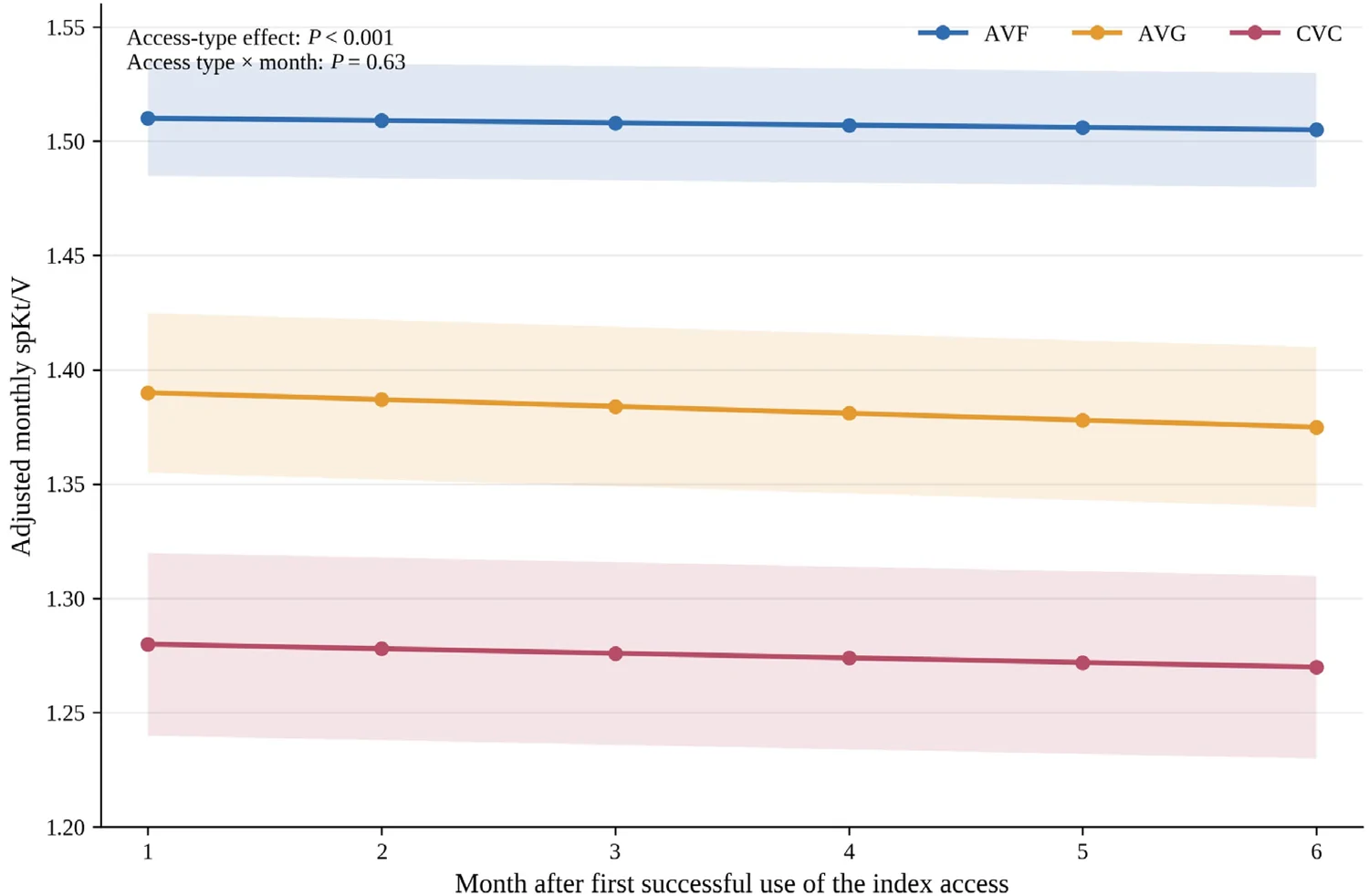
Adjusted single-pool Kt/V trajectories during the first 6 months by index access type. Solid lines show marginal means estimated using the linear mixed-effects model, and shaded bands show 95% confidence intervals (CIs). The model included a patient-specific random intercept, a first-order autoregressive residual correlation structure, access type, month, access type × month, and prespecified pretreatment confounders. AVF, arteriovenous fistula; AVG, arteriovenous graft; CVC, tunneled central venous catheter; spKt/V, single-pool Kt/V.

### 3.4 Access-related complications and incidence rates

The cumulative exposure time of the index access differed among the three groups. Patient proportions and recurrent-event incidence rates were summarized separately. The proportions of patients with infection, a first composite complication, and permanent access failure were highest in the CVC group, intermediate in the AVG group, and lowest in the AVF group (all *P* < 0.001), whereas the proportions with thrombosis and intervention-requiring stenosis did not differ significantly among the groups (both *P* > 0.05). CVC-related bloodstream infections were reported only when the confirmation criteria were met and were applicable only to the CVC group. After individual access-exposure time was taken into account, the incidence rates of infection, thrombosis, intervention-requiring stenosis, all recurrent composite complications, and permanent access failure over the full follow-up differed among the groups (all *P* < 0.001), with the highest overall event burden in the CVC group (Table 3; Figure 3).

**Table 3:** Access-related complications, permanent failure, and access survival from first successful use.

Outcome	AVF group	AVG group	CVC group	Statistical comparison
Index access exposure time, access-years	542.95	187.99	98.73	—
Patients with infection, *n* (%)	6 (3.8)	7 (10.4)	20 (32.3)	<0.001
Infection events; rate per 100 access-years (95% CI)	6; 1.11 (0.41–2.41)	8; 4.26 (1.84–8.39)	28; 28.36 (18.85–40.99)	<0.001
Confirmed CVC-related bloodstream infection events; rate per 100 catheter-years (95% CI)	N/A	N/A	15; 15.19 (8.50–25.06)	—
Patients with thrombosis, *n* (%)	13 (8.3)	13 (19.4)	8 (12.9)	0.063
Thrombosis events; rate per 100 access-years (95% CI)	15; 2.76 (1.55–4.56)	17; 9.04 (5.27–14.48)	10; 10.13 (4.86–18.63)	<0.001
Patients with intervention-requiring stenosis, *n* (%)	21 (13.5)	18 (26.9)	12 (19.4)	0.054
Stenosis events; rate per 100 access-years (95% CI)	27; 4.97 (3.28–7.24)	25; 13.30 (8.61–19.63)	16; 16.21 (9.26–26.32)	<0.001
Patients with a first composite complication, *n* (%)	34 (21.8)	29 (43.3)	35 (56.5)	<0.001
All recurrent composite complications; rate per 100 access-years (95% CI)	48; 8.84 (6.52–11.72)	50; 26.60 (19.74–35.07)	54; 54.69 (41.09–71.36)	<0.001
Permanent failure events within 12 months, *n*	9	10	24	<0.001
12-month Kaplan–Meier access survival from first successful use, % (95% CI)	94.0 (88.8–96.8)	84.7 (73.4–91.5)	57.4 (43.4–69.1)	log-rank *P* < 0.001
24-month Kaplan–Meier access survival from first successful use, % (95% CI)	91.1 (85.2–94.7)	74.7 (62.0–83.7)	40.7 (27.3–53.6)	log-rank *P* < 0.001
Permanent access failure over the full follow-up; rate per 100 access-years (95% CI)	28; 5.16 (3.43–7.45)	35; 18.62 (12.97–25.89)	45; 45.58 (33.25–60.99)	<0.001
Median access survival from first successful use, months	Not reached	44.3	17.5	—

Patient proportions are the number of patients with at least one corresponding event during follow-up divided by the total number of patients in that group. The incidence rate was calculated as the total number of events divided by individual index access-exposure years and multiplied by 100; 95% confidence intervals (CIs) were estimated using the exact Poisson method. Between-group incidence rates were compared using patient-level negative binomial regression with cumulative event count as the outcome, log(individual access-exposure years) as the offset, and robust standard errors. Patient proportions with permanent access failure were compared using the Pearson *χ*^2^ test, and time-to-event outcomes using Kaplan–Meier methods and the log-rank test. Access-survival estimates were measured from first successful use of the index access and therefore apply only to accesses that had reached successful clinical use. All *P* values are two-sided.

AVF, arteriovenous fistula; AVG, arteriovenous graft; CVC, tunneled central venous catheter; CRBSI, catheter-related bloodstream infection; *n*, number of patients; N/A, not applicable.

**Figure 3:**
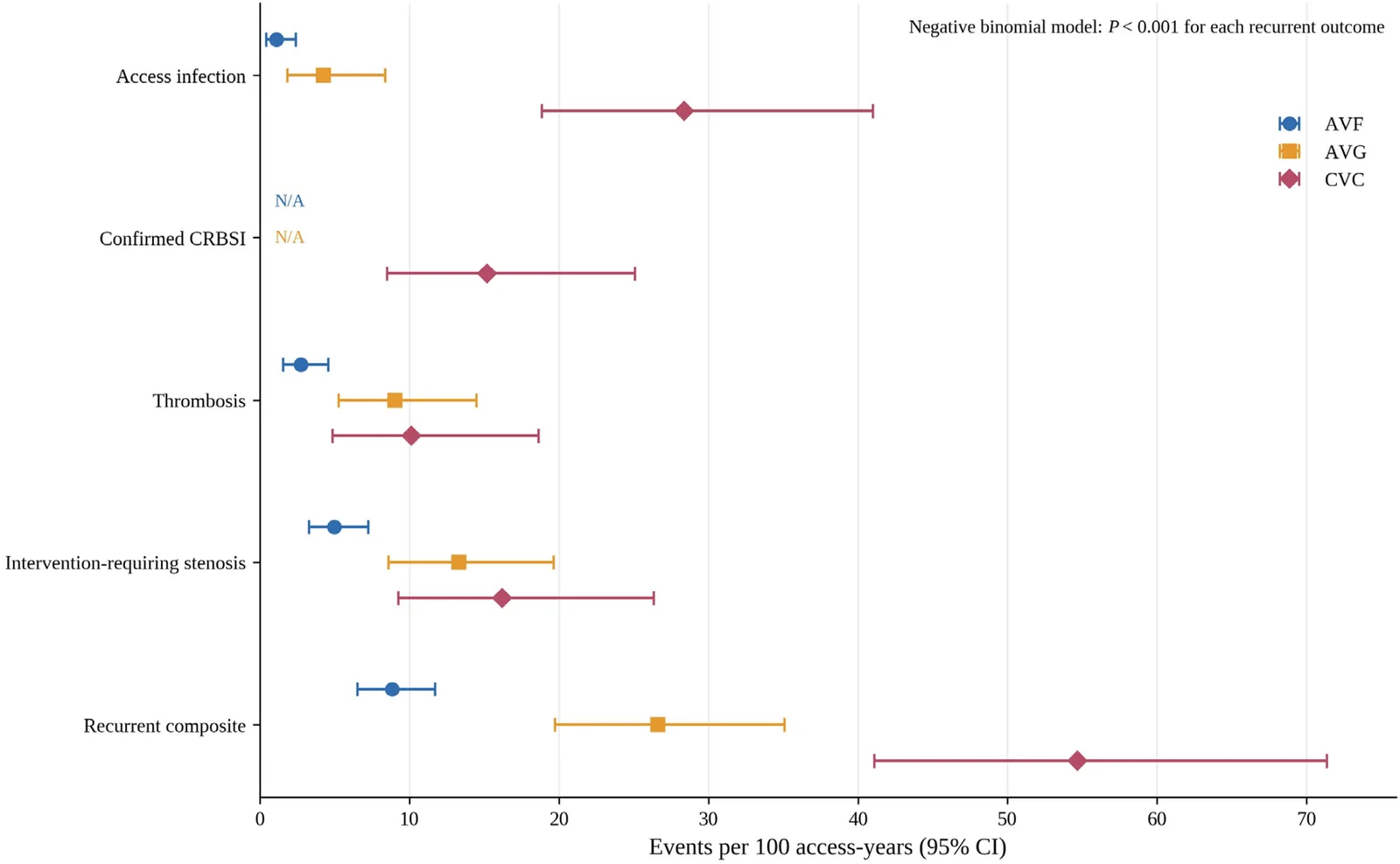
Incidence rates of access-related complications by index access type. Point estimates represent events per 100 access-years, and error bars represent exact Poisson 95% confidence intervals (CIs). Groups were compared using patient-level negative binomial regression with cumulative event count as the outcome, log(individual access-exposure years) as the offset, and robust standard errors. AVF, arteriovenous fistula; AVG, arteriovenous graft; CVC, tunneled central venous catheter; CRBSI, catheter-related bloodstream infection.

### 3.5 Multivariable analyses

In the primary linear mixed-effects model, AVF and AVG were both associated with higher monthly spKt/V than CVC, whereas BMI and diabetes were associated with lower spKt/V. Month, access type ×month, age, sex, cardiovascular disease, dialysis vintage, Kru, history of urgent-start dialysis, previous access attempts, and vascular mapping were not significantly associated with spKt/V (all *P* > 0.05) (Table 4). After achieved treatment parameters were added, the effect estimates for AVF and AVG were attenuated but remained directionally consistent. Actual treatment duration and blood pump flow were positively associated with spKt/V, whereas recirculation and missed dialysis were negatively associated with spKt/V; dialyzer membrane area was not significantly associated with spKt/V (Supplementary Table S2). Repeated binary-outcome models for monthly spKt/V ≥1.2, spKt/V ≥1.4, and URR ≥65% all showed higher odds of threshold attainment for AVF and AVG than for CVC (all *P* < 0.001) (Supplementary Table S3).

**Table 4:** Primary linear mixed-effects model for monthly single-pool Kt/V (spKt/V).

Variable	*β*	95% CI	*P* value
AVF vs. CVC	0.23	0.17–0.29	<0.001
AVG vs. CVC	0.11	0.04–0.18	0.002
Month, per 1-month increase	−0.002	−0.006–0.002	0.327
AVF × month	0.001	−0.003–0.005	0.624
AVG × month	−0.001	−0.006–0.004	0.695
Age, per 10-year increase	−0.01	−0.03–0.01	0.327
Male sex	0.01	−0.03–0.05	0.624
BMI, per 5 kg/m^2^ increase	−0.03	−0.05 to −0.01	0.003
Diabetes	−0.05	−0.09 to −0.01	0.014
Cardiovascular disease	−0.02	−0.06–0.02	0.327
Dialysis vintage, per 12-month increase	0.01	−0.001–0.021	0.075
Kru, per 1 mL/min/1.73 m^2^ increase	0.01	−0.01–0.03	0.327
History of urgent-start dialysis	−0.03	−0.08–0.02	0.240
Previous AVF or AVG attempt	−0.02	−0.06–0.02	0.327
Vascular mapping suitable for native AVF	0.02	−0.03–0.07	0.433

β is the adjusted mean difference in monthly single-pool Kt/V (spKt/V) when other covariates are held constant; positive values indicate a value higher than that of the reference group. The model used a patient-specific random intercept and a first-order autoregressive correlation structure [AR(1)], with month centered at 0 at the first measurement. Fixed effects included access type, month, access type × month, and prespecified pretreatment confounders. Reference categories were CVC, female sex, no diabetes, no cardiovascular disease, nonurgent-start dialysis, no previous AVF or AVG creation attempt, and vascular mapping unsuitable for native AVF creation. Marginal *R*^2^ = 0.43, conditional *R*^2^ = 0.70, and intraclass correlation coefficient (ICC) = 0.47. *P* values are from Wald tests and are two-sided.

AVF, arteriovenous fistula; AVG, arteriovenous graft; CVC, tunneled central venous catheter; BMI, body mass index; Kru, residual kidney urea clearance standardized to a body surface area of 1.73 m^2^; CI, confidence interval.

In the Cox model for the first composite complication, AVF was associated with a lower hazard than CVC, whereas the estimate for AVG was not statistically significant. Diabetes and longer dialysis vintage were associated with a higher hazard. Age, sex, BMI, cardiovascular disease, Kru, history of urgent-start dialysis, previous access attempts, and vascular mapping were not significantly associated with the outcome (all *P* > 0.05) (Table 5). The proportional hazards assumption was satisfied (global *P* = 0.480), the Harrell C statistic was 0.75, and the maximum VIF was 2.7.

**Table 5:** Cox proportional hazards model for the first composite complication (98 first events).

Variable	HR	95% CI	*P* value
AVF vs. CVC	0.39	0.23–0.66	<0.001
AVG vs. CVC	0.72	0.42–1.24	0.234
Age, per 10-year increase	1.07	0.91–1.26	0.415
Male sex	1.11	0.73–1.69	0.626
BMI, per 5 kg/m^2^ increase	1.05	0.87–1.27	0.613
Diabetes	1.58	1.04–2.40	0.032
Cardiovascular disease	1.31	0.85–2.02	0.221
Dialysis vintage, per 12-month increase	1.09	1.03–1.16	0.004
Kru, per 1 mL/min/1.73 m^2^ increase	0.93	0.79–1.10	0.390
History of urgent-start dialysis	1.34	0.80–2.24	0.265
Previous AVF or AVG attempt	1.43	0.95–2.17	0.090
Vascular mapping suitable for native AVF	0.88	0.54–1.43	0.607

The outcome was the composite of the first infection, thrombosis, or intervention-requiring stenosis from time zero, with 98 first events. HR is the adjusted hazard ratio estimated using the Cox proportional hazards model; HR < 1 indicates a lower hazard than the reference group. Reference categories were CVC, female sex, no diabetes, no cardiovascular disease, nonurgent-start dialysis, no previous AVF or AVG creation attempt, and vascular mapping unsuitable for native AVF creation. The proportional hazards assumption was assessed using Schoenfeld residuals, discrimination using the Harrell C statistic, and collinearity using the variance inflation factor (VIF). *P* values are from Wald tests and are two-sided.

AVF, arteriovenous fistula; AVG, arteriovenous graft; CVC, tunneled central venous catheter; BMI, body mass index; Kru, residual kidney urea clearance standardized to a body surface area of 1.73 m^2^; HR, hazard ratio; CI, confidence interval.

### 3.6 Access survival from first successful use and sensitivity analyses

Among accesses that had reached successful clinical use, permanent access failure within 12 months was most frequent in the CVC group, intermediate in the AVG group, and least frequent in the AVF group (*P* < 0.001). Kaplan–Meier access survival measured from first successful use at 12 and 24 months was highest in the AVF group, intermediate in the AVG group, and lowest in the CVC group. Median observed access survival from first successful use was not reached in the AVF group and was progressively shorter in the AVG and CVC groups; the overall log-rank *P* < 0.001. Crude 12-month failure proportions and Kaplan–Meier failure probabilities were reported separately to account for nonfailure censoring before 12 months (Table 3; Figure 4).

**Figure 4:**
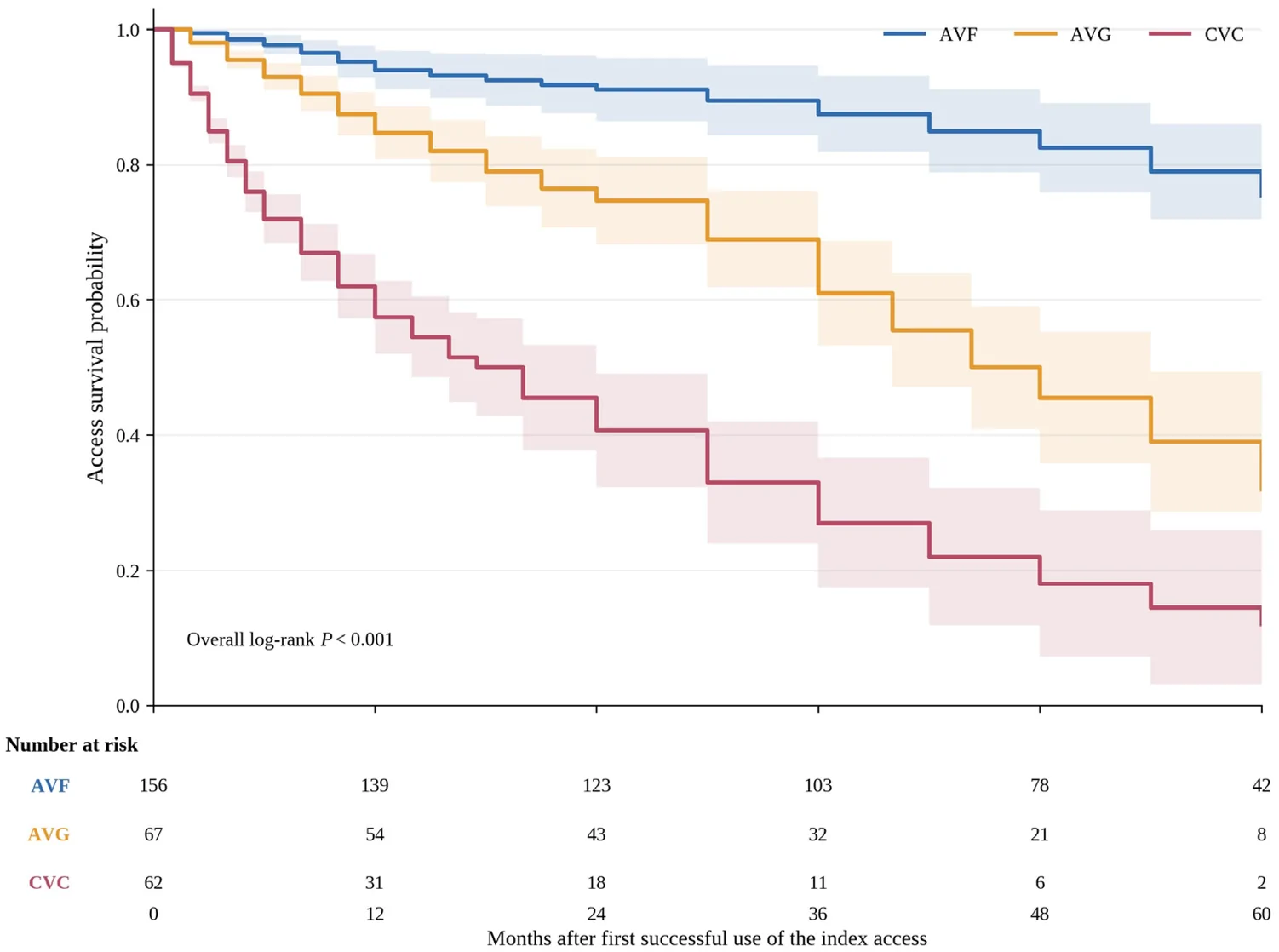
Kaplan–Meier access-survival curves from first successful use by index access type. Time zero was the date on which the index access first successfully supported the prescribed dialysis treatment. Accordingly, the curves describe observed survival only among accesses that had reached successful clinical use and do not include AVF maturation failure or early AVG nonuse before first successful use. Permanent access failure was the event; death, kidney transplantation, transfer or loss to follow-up, planned conversion without failure, and the study end date were censoring events. Solid lines show Kaplan–Meier estimates, and shaded bands show 95% confidence intervals (CIs). Numbers at risk at 0, 12, 24, 36, 48, and 60 months are shown below the plot. Death, kidney transplantation, and planned conversion were treated as competing events in the Fine-Gray sensitivity analysis. Overall log-rank *P* < 0.001. AVF, arteriovenous fistula; AVG, arteriovenous graft; CVC, tunneled central venous catheter.

After three-category propensity-score overlap weighting, the absolute standardized differences for all pretreatment covariates were <0.10. The directions of effect in analyses restricted to complete baseline covariates, complete 6-month trajectories, inverse-probability-of-observation weighting, the subgroup with 24 h urine volume < 200 mL, inclusion of log₂(first-successful-use interval + 1 d), restriction according to the primary CVC indication, the Fine-Gray competing-risk analysis, and ridge-penalized Cox regression were consistent with those of the primary analysis. In the extended model, the interval to first successful use was not significantly associated with monthly spKt/V (*β* = 0.006, 95% CI: −0.006–0.018; *P* = 0.327) or the hazard of the first composite complication (HR = 0.98, 95% CI: 0.91–1.05; *P* = 0.580) (Supplementary Table S4).

## 4 Discussion

Using the first successful use of the index access as time zero, this study compared the associations of AVF, AVG, and tunneled CVC use with repeated dialysis adequacy during the first 6 months, recurrent complications according to access-exposure time, and access survival. AVF use was associated with higher monthly spKt/V and a lower hazard of the first composite complication. AVG use was associated with higher adequacy than CVC use, although the difference in complication hazard was not statistically significant. The CVC group had the greatest burden of infection and permanent access failure. Among accesses that had reached successful clinical use, AVFs showed longer observed access survival than AVGs and CVCs. Previous evidence linking Kt/V trajectories with clinical outcomes supports the use of repeated measurements to characterize long-term dialysis dose (20). Differences in adequacy for AVF and AVG persisted after adjustment for pretreatment factors, but the effect estimates were attenuated after actual treatment duration, blood pump flow, and recirculation were added, suggesting that the association between access type and adequacy may partly reflect differences in achievable treatment delivery. Data-driven adequacy prediction likewise identifies blood flow, treatment duration, and body size as key determinants (21).

The CVC group had greater preservation of residual kidney function, consistent with its shorter dialysis vintage and higher prevalence of urgent-start dialysis. spKt/V and URR reflect urea clearance delivered by dialysis, whereas Kru reflects continuing native-kidney clearance of water and solutes; these two sources of clearance should be considered separately when interpreting adequacy (22). The association between residual kidney function and mortality indicates that failure to account for Kru when comparing access types may incorrectly attribute the physiological advantage of the early dialysis period to the access itself (23). Kru was included in the primary models, and the direction of the findings was confirmed in the subgroup with 24 h urine volume <200 mL. The lower interdialytic weight-gain percentage and ultrafiltration rate in the CVC group were also consistent with its greater residual urine volume. Under selected conditions, patients with preserved residual kidney function may maintain solute and volume control with different dialysis frequencies, indicating that adequacy assessment should integrate actual treatment frequency, treatment duration, kidney clearance, and volume management (24).

The incidence rate of access-related infection was markedly higher in the CVC group. National surveillance data show a close association between catheter exposure and hemodialysis-associated Staphylococcus aureus bloodstream infection, with healthcare access and social factors also contributing (25). Catheter dwell time, diabetes, previous infection, and care processes are important risk factors for catheter-related bloodstream infection (26). Because the CVC group in this study had higher proportions of diabetes and urgent-start dialysis, the observed differences in infection reflect both catheter characteristics and patient selection. Tunneled CVCs may improve some short-term outcomes compared with nontunneled catheters, but risks of infection, dysfunction, and central venous disease remain (27). Catheter-locking strategies can influence infectious and thrombotic events, but potential benefits must be balanced against bleeding, antimicrobial resistance, and cost (28). These findings support reducing avoidable catheter exposure and strengthening standardized catheter care.

The AVG group had higher incidence rates of thrombosis and intervention-requiring stenosis than the AVF group, reflecting the additional maintenance burden associated with prosthetic material, anastomotic hemodynamics, and neointimal hyperplasia. Pharmacological, interventional, and device-based strategies continue to evolve, but early recognition of functional decline and timely treatment of stenosis remain central to maintaining patency (29). The timing of access creation directly affects catheter exposure at dialysis initiation. Earlier creation does not guarantee that every patient will have a usable AVF or AVG when dialysis begins, because the rate of kidney function decline and maturation failure alter the expected benefit (30). We therefore reported the history of urgent-start dialysis, previous access attempts, vascular mapping, and the interval from creation or placement to first successful use. Vascular access management has shifted from a single access hierarchy to kidney failure life planning and serial decision-making. Overemphasis on a single access metric may increase unused procedures, repeated interventions, or treatment burdens that do not align with patient goals (31). Long-term outcome data indicate that the associations of access type with survival and failure are influenced by age, comorbidity, access transitions, and treatment phase (32). These associations should therefore be interpreted within those contexts when individualized, patient-centered access decisions are made.

Recent clinical outcome data generally show a lower infection burden with AVF, whereas AVG has practical value when a permanent cannulatable access is needed more rapidly (33). Access selection should consider the anticipated timing of dialysis initiation, vascular conditions, previous failures, life expectancy, and future treatment pathways. Patient satisfaction with different access types depends not only on infection and patency but also on cannulation pain, appearance, bathing restrictions, effects on activity, and treatment convenience (34). Catheters and fistulas affect different dimensions of quality of life, and a single survival metric cannot substitute for patient experience (35). Patient-reported outcomes were not measured in this study; clinical decisions should therefore be supplemented by shared decision-making. Health literacy, access-related knowledge, and participation in decision-making complement clinical measures such as patency and infection rates and may help identify patients' actual needs regarding access function and treatment experience (36). Current perspectives emphasize selecting the appropriate access for the appropriate patient at the appropriate time, thereby reducing avoidable catheter exposure while also minimizing ineffective procedures or accesses that fail to mature (37). The present findings support a patient-centered framework and quantify the observational associations of access type with dialysis adequacy and access-related complications. Standardized vascular-access definitions distinguish primary failure before successful use from functional patency after the access becomes usable (38). Consequently, the observed access-survival differences in this study apply only to accesses that had reached successful clinical use and should not be interpreted as overall access-strategy survival or superiority from access creation onward.

This study has several limitations. First, the retrospective design and nonrandom access selection could not fully eliminate unmeasured confounding, and residual confounding between access type and clinical indication may remain. Second, cohort entry began at the first successful use of the index access; the access-survival findings therefore apply only to accesses that had reached successful clinical use and do not capture AVF maturation failure or early AVG nonuse before first successful use. Third, the study was conducted at a single tertiary hospital, and local practices regarding access creation, cannulation, catheter care, and interventional thresholds may limit external applicability. Fourth, some data on Kru, vascular mapping, and treatment parameters were missing; although multiple imputation and complete-case analyses were performed, information bias may remain. Fifth, some monthly adequacy measurements during the first 6 months were unavailable because of access failure, planned conversion, hospitalization, or sampling procedures; complete-trajectory and inverse-probability-of-observation weighted analyses reduced but could not fully exclude informative missingness. Sixth, planned conversion and death may have produced informative censoring, which the competing-risk analysis addressed only partially. Seventh, recurrent complications depended on clinical records, and minor events or events managed outside the hospital may have been missed. Eighth, 98 first composite complications occurred; although prespecified Cox modeling and a ridge-penalized sensitivity analysis improved stability, estimates for some covariates may still have been limited by the number of events. Ninth, the study did not include patient-reported outcomes, access-creation costs, or the number of interventions.

## 5 Conclusion

In this study population, AVF use was associated with higher dialysis adequacy and a lower burden of access-related complications. Among accesses that had reached successful clinical use, AVFs showed longer observed access survival than AVGs and CVCs. Dialysis adequacy was higher with AVG than with CVC, whereas the estimated hazard of the first composite complication did not differ clearly between these access types. CVCs were used mainly in patients requiring urgent-start dialysis, patients with unfavorable vascular or systemic conditions, patients with exhausted previous access options, or patients whose permanent access was not yet usable, and CVC use was accompanied by greater burdens of infection and access failure. Vascular access decisions should integrate the anticipated timing of dialysis, vascular anatomy, comorbidity, residual kidney function, previous access history, life expectancy, future treatment pathways, and patient preferences.

## Data Availability

The data that support the findings of this study are available from the corresponding author upon reasonable request.
