# Supplementary material for: Association of hemodialysis vascular access type with dialysis adequacy and access-related outcomes in patients receiving maintenance hemodialysis
Source: Front Med (Lausanne). 2026 Oct 7;13:1890240. doi: 10.3389/fmed.2026.1890240 (PMC13683122; doi:10.3389/fmed.2026.1890240)
Supplement: Supplementary file 1 [file Supplementaryfile1.docx]

# **Supplementary Material**

**Supplementary Table 1. Primary Indications for CVC Use**

| Primary indication for CVC use | n (%) |
| --- | --- |
| Urgent-start dialysis with no immediately usable AVF or AVG | 24 (38.7) |
| Vascular anatomy or systemic condition unsuitable for timely AVF or AVG creation or use | 16 (25.8) |
| Repeated failure of previous permanent access or exhaustion of usable sites | 12 (19.4) |
| Waiting for a created AVF or AVG to mature | 7 (11.3) |
| Informed patient choice or other reasons | 3 (4.8) |
| Total | 62 (100.0) |

**Note:** Each patient with a CVC was assigned to one primary indication according to a prespecified mutually exclusive hierarchy based on dialysis-initiation records, vascular mapping, access-surgery or interventional records, and patient-decision records. Data are presented as number (percentage), and no between-group inferential test was performed. AVF, arteriovenous fistula; AVG, arteriovenous graft; CVC, tunneled central venous catheter; n, number of patients.

**Supplementary Table 2. Explanatory Linear Mixed-Effects Model Adjusted for Treatment-Delivery Parameters**

| Variable | *β* | 95% CI | *P* value |
| --- | --- | --- | --- |
| AVF vs CVC | 0.18 | 0.12–0.24 | <0.001 |
| AVG vs CVC | 0.08 | 0.01–0.15 | 0.025 |
| Actual treatment duration, per 30 min | 0.05 | 0.03–0.07 | <0.001 |
| Blood pump flow, per 50 mL/min | 0.04 | 0.02–0.06 | <0.001 |
| Access recirculation, per 5% | −0.03 | −0.05–−0.01 | 0.003 |
| Dialyzer membrane area, per 0.2 m² | 0.01 | −0.01–0.03 | 0.327 |
| Missed dialysis in the current month | −0.06 | −0.11–−0.01 | 0.019 |

**Note:** *β* is the adjusted mean difference in monthly single-pool Kt/V (spKt/V). In addition to the pretreatment confounders included in the primary model, this explanatory linear mixed-effects model included actual treatment duration, blood pump flow, access recirculation, dialyzer membrane area, and missed dialysis in the current month to describe the residual association after adjustment for treatment-delivery factors. *P* values are from Wald tests and are two-sided. AVF, arteriovenous fistula; AVG, arteriovenous graft; CVC, tunneled central venous catheter; CI, confidence interval.

**Supplementary Table 3. Generalized Linear Mixed Models for Monthly Dialysis-Adequacy Thresholds**

| Outcome | Comparison | aOR | 95% CI | *P* value |
| --- | --- | --- | --- | --- |
| Monthly spKt/V ≥ 1.2 | AVF vs CVC | 7.45 | 4.86–11.42 | <0.001 |
| Monthly spKt/V ≥ 1.2 | AVG vs CVC | 2.88 | 1.85–4.49 | <0.001 |
| Monthly spKt/V ≥ 1.4 | AVF vs CVC | 6.31 | 4.21–9.45 | <0.001 |
| Monthly spKt/V ≥ 1.4 | AVG vs CVC | 2.39 | 1.58–3.61 | <0.001 |
| Monthly URR ≥ 65% | AVF vs CVC | 7.02 | 4.68–10.54 | <0.001 |
| Monthly URR ≥ 65% | AVG vs CVC | 2.48 | 1.64–3.74 | <0.001 |

**Note:** Monthly binary outcomes were analyzed using generalized linear mixed-effects models with a patient-specific random intercept and logit link. aOR is the adjusted odds ratio relative to CVC; adjustment variables were the same as those in the primary linear mixed-effects model for monthly spKt/V. An aOR > 1 indicates higher odds of attaining the corresponding threshold. CVC was the reference group. *P* values are from Wald tests and are two-sided. AVF, arteriovenous fistula; AVG, arteriovenous graft; CVC, tunneled central venous catheter; spKt/V, single-pool Kt/V; URR, urea reduction ratio; aOR, adjusted odds ratio; CI, confidence interval.

**Supplementary Table 4. Robustness and Sensitivity Analyses**

| Sensitivity analysis | AVF vs CVC | AVG vs CVC | Conclusion |
| --- | --- | --- | --- |
| Propensity-score overlap weighting: monthly spKt/V | 0.21 (0.15–0.27), *P* < 0.001 | 0.10 (0.03–0.17), *P* = 0.005 | Direction consistent |
| Complete cases for baseline covariates: monthly spKt/V | 0.22 (0.16–0.28), *P* < 0.001 | 0.10 (0.03–0.17), *P* = 0.004 | Direction consistent |
| Complete 6-month trajectory: monthly spKt/V | 0.22 (0.16–0.28), *P* < 0.001 | 0.10 (0.03–0.17), *P* = 0.004 | Direction consistent |
| Inverse-probability-of-observation weighting: monthly spKt/V | 0.22 (0.16–0.29), *P* < 0.001 | 0.10 (0.03–0.18), *P* = 0.006 | Direction consistent |
| Subgroup with 24-h urine volume < 200 mL: monthly spKt/V | 0.20 (0.13–0.27), *P* < 0.001 | 0.09 (0.01–0.17), *P* = 0.029 | Direction consistent |
| Including log₂(first-successful-use interval + 1 d): monthly spKt/V | 0.22 (0.16–0.28), *P* < 0.001 | 0.10 (0.03–0.17), *P* = 0.004 | Direction consistent; interval *β* =0.006 (−0.006–0.018), *P* = 0.327 |
| Restriction according to the primary CVC indication (CVC, n = 52): monthly spKt/V | 0.22 (0.16–0.29), *P* < 0.001 | 0.10 (0.03–0.18), *P* = 0.007 | Direction consistent |
| Complete cases for baseline covariates: first composite complication HR | 0.41 (0.24–0.70), *P* < 0.001 | 0.75 (0.43–1.31), *P* = 0.313 | Direction consistent |
| Including log₂(first-successful-use interval + 1 d): first composite-complication HR | 0.40 (0.23–0.68), *P* < 0.001 | 0.74 (0.43–1.28), *P* = 0.286 | Direction consistent; interval HR = 0.98 (95% CI 0.91–1.05), *P* = 0.580 |
| Restriction according to the primary CVC indication (CVC, n = 52): first composite-complication HR | 0.41 (0.24–0.70), *P* < 0.001 | 0.76 (0.44–1.32), *P* = 0.329 | Direction consistent |
| Fine-Gray permanent-access-failure sHR | 0.34 (0.21–0.56), *P* < 0.001 | 0.67 (0.41–1.10), *P* = 0.114 | AVF association retained |
| Ridge-penalized Cox model: first composite complication HR | 0.43 (0.28–0.67), *P* < 0.001 | 0.76 (0.49–1.18), *P* = 0.223 | Direction consistent |

**Note:** Continuous adequacy outcomes are reported as adjusted *β* coefficients, the first composite complication as adjusted HRs, and the competing-risk analysis as subdistribution hazard ratios (sHRs); values in parentheses are 95% confidence intervals (CIs). Propensity-score overlap weighting used a multinomial propensity score. Complete-case analyses included only patients without missing covariates. The Fine-Gray model treated death, kidney transplantation, and planned conversion without failure as competing events. Ridge-penalized Cox regression assessed estimate stability under event-count constraints. All *P* values are two-sided. AVF, arteriovenous fistula; AVG, arteriovenous graft; CVC, tunneled central venous catheter; spKt/V, single-pool Kt/V; HR, hazard ratio.
